# Regulators of glucose uptake in thyroid cancer cell lines

**DOI:** 10.1186/s12964-020-00586-x

**Published:** 2020-06-03

**Authors:** Shabnam Heydarzadeh, Ali Asghar Moshtaghie, Maryam Daneshpoor, Mehdi Hedayati

**Affiliations:** 1 Department of Biochemistry, School of Biological Sciences, Falavarjan Branch Islamic Azad University, Isfahan, Iran; 2 Cellular and Molecular Endocrine Research Center, Research Institute for Endocrine Sciences, Shahid Beheshti University of Medical Sciences, Tehran, Iran

**Keywords:** Thyroid cancer, Glucose uptake, Regulator, Glucose transporter

## Abstract

**Abstract:**

Thyroid cancer is the most common sort of endocrine-related cancer with more prevalent in women and elderly individuals which has quickly widespread expansion in worldwide over the recent decades. Common features of malignant thyroid cells are to have accelerated metabolism and increased glucose uptake to optimize their energy supply which provides a fundamental advantage for growth. In tumor cells the retaining of required energy charge for cell survival is imperative, indeed glucose transporters are enable of promoting of this task. According to this relation it has been reported the upregulation of glucose transporters in various types of cancers. Human studies indicated that poor survival can be occurred following the high levels of GLUT1 expression in tumors. GLUT-1 and GLUT3 are the glucose transporters which seems to be mainly engaged with the oncogenesis of thyroid cancer and their expression in malignant tissues is much more than in the normal one. They are promising targets for the advancement of anticancer strategies. The lack of oncosuppressors have dominant effect on the membrane expression of GLUT1 and glucose uptake. Overexpression of hypoxia inducible factors have been additionally connected with distant metastasis in thyroid cancers which mediates transcriptional regulation of glycolytic genes including GLUT1 and GLUT3. Though the physiological role of the thyroid gland is well illustrated, but the metabolic regulations in thyroid cancer remain evasive. In this study we discuss proliferation pathways of the key regulators and signaling molecules such as PI3K-Akt, HIF-1, MicroRNA, PTEN, AMPK, BRAF, c-Myc, TSH, Iodide and p53 which includes in the regulation of GLUTs in thyroid cancer cells. Incidence of deregulations in cellular energetics and metabolism are the most serious signs of cancers. In conclusion, understanding the mechanisms of glucose transportation in normal and pathologic thyroid tissues is critically important and could provide significant insights in science of diagnosis and treatment of thyroid disease.

Video Abstract

**Graphical abstract:**

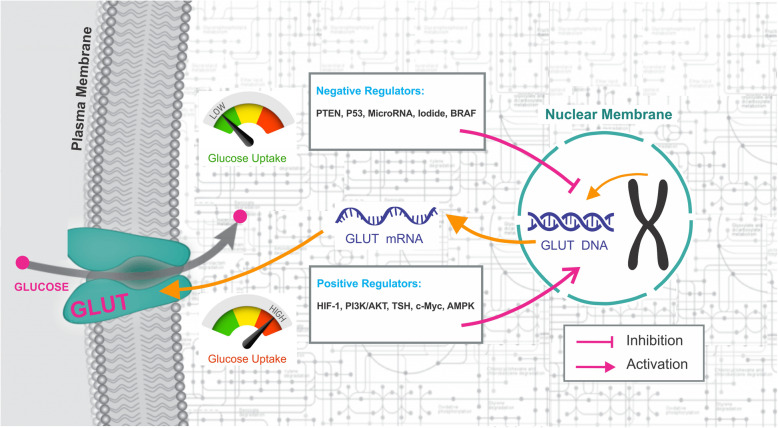

## Key points

- The up regulation of Glucose transporters (GLUTs) has been reported in thyroid cancer which has been shown to be an index of aggressiveness and loss of tumor differentiation
- The identification of different regulators and signaling molecules which involve in the expression and subcellular distribution of several GLUTs can aid in the diagnosis and clinical management of thyroid cancer
- GLUTs as rate-limiting steps in glucose metabolism of cancer cells and regulators of glucose uptake pathway are promising targets for the development of anticancer strategies

## Background

In glucose metabolism, the translocation of glucose across the plasma membrane known as the rate limiting step happens by carriers belonging to the facilitative glucose transporter (GLUT) and the sodium-coupled glucose co-transporter (SGLT) proteins families. While the SGLTs require energy to the task of glucose transportation, the GLUTs allow the transport of glucose down its concentration gradient without energy dependence [1]. GLUT1 is one of the fourteen isoforms of GLUTs with great affinity for glucose which represents unusual overexpression in the plasma membrane [2, 3]. High expression of GLUT1, positively correlate with the proliferative index and equates to the malignant characteristic. In this condition scientists have poor foresight in various types of tumors, including prostate [4], thyroid [5, 6], colon [7, 8], melanoma [9], liver [10], breast [11, 12], and ovary [13, 14].

Most cancer cells alter cellular metabolisms because of achieving high proliferation rates and this can be lead to constitutive stress metabolic phenotype. Tumor cells are capable of switching metabolism from oxidative to the glycolytic phenotype. It is called Warburg effect which is tumor-specific metabolic characteristic and a key metabolic hallmark. Researches around tumor metabolism show that through the alteration of cellular metabolism in which glycolysis and glutaminolysis are up regulated, it is necessary to maintain rapid cell proliferation, tumor progression, and resistance to cell death [15]. Glucose transportation in neoplastic cells occurs across the plasma membrane which is the first rate-limiting steps of glucose metabolism. There is evidence that GLUT1 reduction can suppress cell proliferation, therefore the regulation of glucose transporter expression and activity have significant effect on the supply of glucose in cancer cells [16]. Several studies have demonstrated the usefulness of glucose transporter 1 (GLUT 1) immunohistochemistry in cancer cell researches [17–19]. Overexpression of GLUT-1 on the cell membrane is exactly associated with the rate of cell differentiation and greater biological aggressiveness of thyroid cancer being found more in anaplastic thyroid cancers than in well-differentiated forms. GLUT-1 are localized on cell membrane and the expression of these transporters can be evaluated by positron emission tomography (PET) [20].

A defining characteristic of thyroid cancer cells is their strong ability to take up enormous amounts of glucose compared to normal thyroid tissue. Malignant cells rewire their metabolism through enhancement of glucose uptake for promotion of cell growth and survival. Tumor cells enhance glucose uptake across the plasma membrane via induction of a family of facilitative glucose transporter proteins (GLUTs), which classified regarding their tissue-specific distribution and different affinities for glucose and remarkably different transport capacities. In most cases thyroid cancer cells frequently show overexpression of especially the hypoxia-responsive GLUT1 and GLUT3 proteins. Malignant cells characteristically have a reduced ability to use oxidative metabolism, and instead aerobic glycolysis increased rapidly and oxidative phosphorylation remained stable. Increased glycolysis is the main source of energy supply in cancer cells but, due to the lower energy yield of the glycolytic pathway, malignant cells show an increased rate of glucose transport across the plasma membrane to compensate the acquired energy [21–25].

Recently the tumor differentiation relationship with glucose metabolism in the thyroid cancer is evaluated. The glucose metabolic profiles have complex association with tumor differentiation in well differentiated thyroid cancer (Papillary thyroid cancer) and poorly differentiated thyroid cancer (Anaplastic thyroid cancer). According to the Suh H. Y. et al. analysis based on the genetic mutation status, metabolic profiles of thyroid cancer were not easily correlated with tumor differentiation. The cellular expression of GLUT was negatively related to tumor differentiation in thyroid cancers. The enrichment of glycolysis was positively correlated with the well-differentiated thyroid cancer, but it has negative association in the case of poorly differentiated thyroid cancer. In papillary thyroid cancer, the glycolysis signature was positively correlated with tumor differentiation score, while the GLUT signature has negative association with tumor differentiation score. On the other hand, in poorly differentiated thyroid cancer, both GLUT and glycolysis signatures have negative correlation with tumor differentiation score. Their results were agreed with the previous studies because poorly differentiated thyroid cancers need higher glucose uptake by representing high GLUT expression. But, glycolysis signatures of thyroid cancers with poor differentiation have conflicting relation between papillary thyroid cancer and anaplastic thyroid cancer. Overall considering that the association between the differentiation and glycolysis might have ‘U shape’ pattern. The reason of different scores for GLUT and glycolysis in papillary thyroid cancer was different BRAFV600E mutation status. BRAFV600E positive PTC have higher GLUT signature and lower glycolysis signature than BRAFV600E negative PTC [26].

Among the glucose transporters, GLUT1, GLUT3, GLUT4, and GLUT10 are the types that detected to be expressed in all thyroid parenchymal cells regardless of histology. Results have been showed that GLUT1 has significantly higher expression in samples of patients with thyroid cancer in comparison with normal and benign tissue samples from the same patient. It has been reported that other GLUTs were not changed in comparison to GLUT1 in pathologic tissues of same patient. These results indicated that GLUT1 is theoretically responsible for the observed enhanced uptake of glucose during carcinogenesis [19, 27]. The overexpression of hexokinase I resulting in increased phosphorylation of intracellular glucose in thyroid tumors has been shown to be a signal of tumor aggressiveness. Tumor differentiation rate in thyroid cancer coordinates with the expression of GLUTs. While poorly differentiated types (anaplastic) have high expression of GLUTs (mainly GLUT-1), conversely, well-differentiated tumors (follicular and papillary) have often poor GLUT-1 expression. It has been reported that GLUT-3 is predominant in papillary thyroid cancer [20, 28]. According to research results, there were positive expression of GLUT-1, GLUT-3, and GLUT-4 in the cytoplasm and/or membrane of papillary thyroid cancer (PTC) cells. In these cells GLUT-3 and GLUT-4 expression was stronger than GLUT-1 expression [29].

There is difference in the expression of hypoxia-related GLUT1 and GLUT3 between benign and malignant neoplasms, as well as non-neoplastic thyroid lesions. The differences in GLUT1 and GLUT3 expression levels are associated with the histological type of thyroid carcinomas. Both hypoxia-related GLUT1 and 3 are involved in the progression of papillary thyroid carcinomas and may be added to a panel of biological thyroid carcinoma markers. There is a close correlation between the overexpression of GLUT1 and GLUT3 proteins and the high levels of GLUT1 and GLUT3 mRNA in the same thyroid cancer specimens. However, in some neoplasm cases the detection of GLUT1 or GLUT3 positive band and the mRNA level was very low. A hypothesis that provides an acceptable explanation for these findings is that the expression of hypoxia-related GLUTs is further influenced by the microenvironment of tumor cells. The deregulation of glucose metabolism in cancer cells is predominantly mediated by oxygen-related transcription factors, such as the hypoxia-inducible factor 1 (HIF-1). HIF-1 induces a number of genes encoding glycolytic enzymes, erythropoietin, the tumor-associated carbonic anhydrases (CAs), CAIX and CAXII, vascular endothelial growth factor (VEGF), as well as the facilitative GLUT isoforms 1 and 3 (GLUT1 and GLUT3). Hypoxia-related GLUTs are characterized by low Km values and high affinity for glucose as compared to other members of the GLUT family [30].

The abnormal surface expression of GLUT proteins and also the activity of GLUT-1 is under the control of multiple signal transduction pathways, including the phosphoinositide 3-kinase (PI3K)/AKT pathway [31]. In thyroid glands, AMPK plays an important physiological role in thyroid iodide uptake and could be involved in some pathophysiological conditions such as tumorigenesis. Recently, there is evidence that AMPK can increases glucose uptake in thyroid cells by increasing both GLUT 1 expression and hexokinase (HK) activity independent of TSH signaling [32]. Loss of expression of the oncosuppressor PTEN, a protein–lipid phosphatase that switches off the AKT pathway, was associated with the surface expression of GLUT1 and increased the possibility of “incidental” detection of thyroid cancer based on FDG–PET [33].

Thyroidectomy (surgical removal of the entire thyroid gland) and radioactive iodine therapy are the routine treatments for patients suffering from thyroid cancer, but it is often not more effective treatment. The recent advancement in molecular-targeted therapies understanding the molecular pathogenesis of thyroid cancer has shown great promise for the development of early diagnosis and appropriate treatment strategies for thyroid cancer. This has basically eventuated from the recognition of molecular alterations [34]. Although the physiological function of the thyroid gland is well defined, its metabolic adaptations remain elusive particularly in thyroid cancer. In this review, we argue recent remarkable progress and main agents which includes in inhibition or stimulation of glucose uptake in thyroid carcinoma that could be helpful for future therapeutic targets in this disease.

### Glucose uptake

Since the early observation by Otto Warburg, it has been well known that tumor cells are characterized by an increased requirement for energy metabolism. According to scientific researches approximately 90% of cancers indicates the phenotype of increased glucose metabolism. Additionally, these cells, have a reduced ability to use mitochondrial oxidation and favor the conversion of pyruvate into lactate, despite the availability of oxygen. Because ATP synthesis is not the top priority of the upregulation of glucose transport. Glycolysis is nearly 18 times less efficient compared to oxidative phosphorylation process so cancer cells require an increased rate of glucose uptake into cells for compensation of the low ATP production [35–37]. Although the Warburg effect was observed more than 80 years ago, but nevertheless its interpretation is argumentative and evolving. Cancer cells do not desire to convert all the glucose achieving from the upregulated transportation to pyruvate, but instead diverts some glucose metabolic intermediates to pentose phosphate pathway (PPP) which is a metabolic pathway branched off from glycolysis provides metabolic intermediates for synthesis of biomass [38–40]. Currently, clinical and basic science studies have shown that Warburg effect is a very potential and smart cancer research area [41]. Targeting glucose metabolism and transportation, have been suggested as a beneficial purpose for cancer therapeutic intervention [37, 42, 43]. In addition to higher potentials for invasiveness and metastasis [44], the glycolytic switch in cancer also increases cancer’s sensitivity to external interference because of their higher dependence on aerobic glycolysis [45–47]. The exploration of GLUTs inhibitors might be illuminate the development of drugs that could be exerted as anticancer agents, probably beside conventional chemotherapies or new immunotherapies to pursue in future studies [48]. There is ensuring evidence that the expression, activity and Intracellular traffic of GLUTs as biomarkers of malignancy are regulated by various signaling molecules and pathways.

### The relationship between FDG-PET, GLUT, hexokinase and differentiation

The application of ^18^F-fluorodeoxyglucose positron emission tomography (FDG-PET) for detection of thyroid cancer patients has come into the spotlight recently. FDG-PET has been identified to display metastasis in ^131^I scan-negative thyroid cancer patients with a high accuracy, which is thought to be related to increased glucose metabolism in poorly differentiated carcinoma. It has proved that FDG avidity has correlation with tumor size, lymph node metastasis, glucose transporter expression and differentiation [49]. Glycolysis activity can be noninvasively evaluated by FDG PET which is associated with FDG retention and be displayed by dual-time FDG PET. Metabolic profiles of thyroid cancer were not easily correlated with tumor differentiation. The cellular expression of GLUT was negatively related to tumor differentiation in thyroid cancers. The enrichment of glycolysis was positively correlated with the well-differentiated thyroid cancer, but it has negative association in the case of poorly differentiated thyroid cancer [26].

FDG, a glucose derivate labelled with F18, showing avidity in cancer types with high tumor related metabolism and upregulation of the glucose transporter system. The role of FDG PET represented by the development of metastatic diseases, which radioiodine therapy was not efficient for them. FDG PET is more sensitive for the patients with an aggressive histological subtype. Highly differentiated thyroid cancer cells still have the sodium-iodide symporter (NIS) expression, which led to iodine uptake in the thyroid. This ability is low for less differentiated cells. On the contrary, FDG is internalised to the cell by a transporter protein (Glut-1), which is overexpressed in malignant cell types. Inverse alterations of either iodine or FDG uptake in metastases are called the flipflop phenomenon [50].

It is commonly accepted that high ^18^F-FDG uptake reflects dedifferentiation of thyroid tumors. Radioiodine-negative metastases display increased glucose uptake more frequently than radioiodine-positive neoplasms. In addition, the survival of patients harboring ^18^F-FDG–positive metastases is significantly shorter than that of cases with ^18^F-FDG–negative metastases. The molecular mechanisms of glucose metabolism in thyroid cancer are not entirely comprehended. It has been proved that ^18^F-FDG uptake can be stimulated by TSH in thyroid cancer tissue in vivo. Although, higher sensitivity of ^18^F-FDG PET for detection of thyroid carcinoma tissue at higher TSH levels has not been proven in clinical experiments. ^18^F-FDG uptake regulation in the thyroid cancer cells is performing by TSH or cAMP or mediated by GLUT-1. This regulation is correlated with high activity of PI3-kinase caused by the active mutated K-ras oncogene. These results indicate that high ^18^F-FDG uptake in thyroid cancer cells observed by PET may be the reflection of intracellular signal transduction cascades stimulation by oncogenes.

It is evident that the specific uptake of this tracer is controlled by the various isoforms of GLUT as well as hexokinase. GLUT-1, GLUT-3, GLUT-4, and GLUT-10 mRNA expression has been detected in normal thyroid tissue. GLUT-1 is the main glucose transporter mediating the transport of ^18^F-FDG within the tumor cell membrane. Although, thyroid cancer cell line ML-1 do not show GLUT-1 expression in their membranes but instead GLUT-3 expression is significantly detected. Ninety-five percent of human follicular carcinomas are deficient for GLUT-1 mRNA. This observation is not completely unexpected and also agrees with results obtained by Ciampi et al., who reported an overexpression of GLUT-3 in other well differentiated follicular thyroid carcinoma cell lines. Phosphorylation is the serious prerequisite for the intracellular trapping of ^18^F-FDG. This phosphorylation is catalyzed by hexokinase. According to immunohistochemical staining results, the ^18^F-FDG retention in thyroid carcinoma tissue is shown to have a correlation with hexokinase I expression [51].

The first key enzyme involved in glycolysis is a hexokinase, so GLUTs and HKs are important for mediating glucose metabolism in tumorigenesis, particularly GLUT-1 and HK-II. Abnormal expression of GLUT-1 and HK-II in many malignant tumors is associated with invasiveness and metastasis of tumors including head and neck cancers such as thyroid carcinoma. Few studies have focused on HKII in head and neck cancers such as thyroid carcinoma. Several studies have shown that GLUT-1 and HK II expression levels are related to tumor FDG uptake, but there are differences among tumor types. The uptake of FDG by some malignant tumors was related to tumor expression levels of GLUT-1 or HK-II, or both; however, uptake of FDG by other tumors was independent of either GLUT-1 or HK-II expression [29]. As mentioned in recent published paper by Suh H. Y. et al. their results showed that in the PTC as the most common thyroid cancer, HK1 was showed increasing level as many previous references. On the other hand, in PDTC, HK1 decreased, but instead HK2 increased [26]. According to Hooft L. et al. research, patients representing cytoplasmic HK I overexpression showed positive ^18^F-FDG PET. Moreover, a positive association was found for cytoplasmic Glut-1 staining. Well differentiated thyroid cancer expressed Glut-1 mostly in the cytoplasm, rather than on the cell membrane. Several studies reported considerable data that Glut-1 expression on the membrane is mainly found around necrotic areas. The yield of ^18^F-FDG PET positivity seems to be predicted by HK I expression. If affirmed in larger studies, their results may have important clinical implications. Different types of cancer indicate a significant association between ^18^F-FDG uptake, Glut-1 and HK II expression. Although HK II was expressed in almost all tumors without a significant relation with ^18^F-FDG uptake. However, correlation of HK I with ^18^F-FDG was identical with experiment on breast cancer cells. The role of the different isozymes of HKs in thyroid cancer is not clearly understood so extra research is required to explain the function of HKs in thyroid cancer [28].

### Negative regulators

#### PTEN

**Fig. 1:**
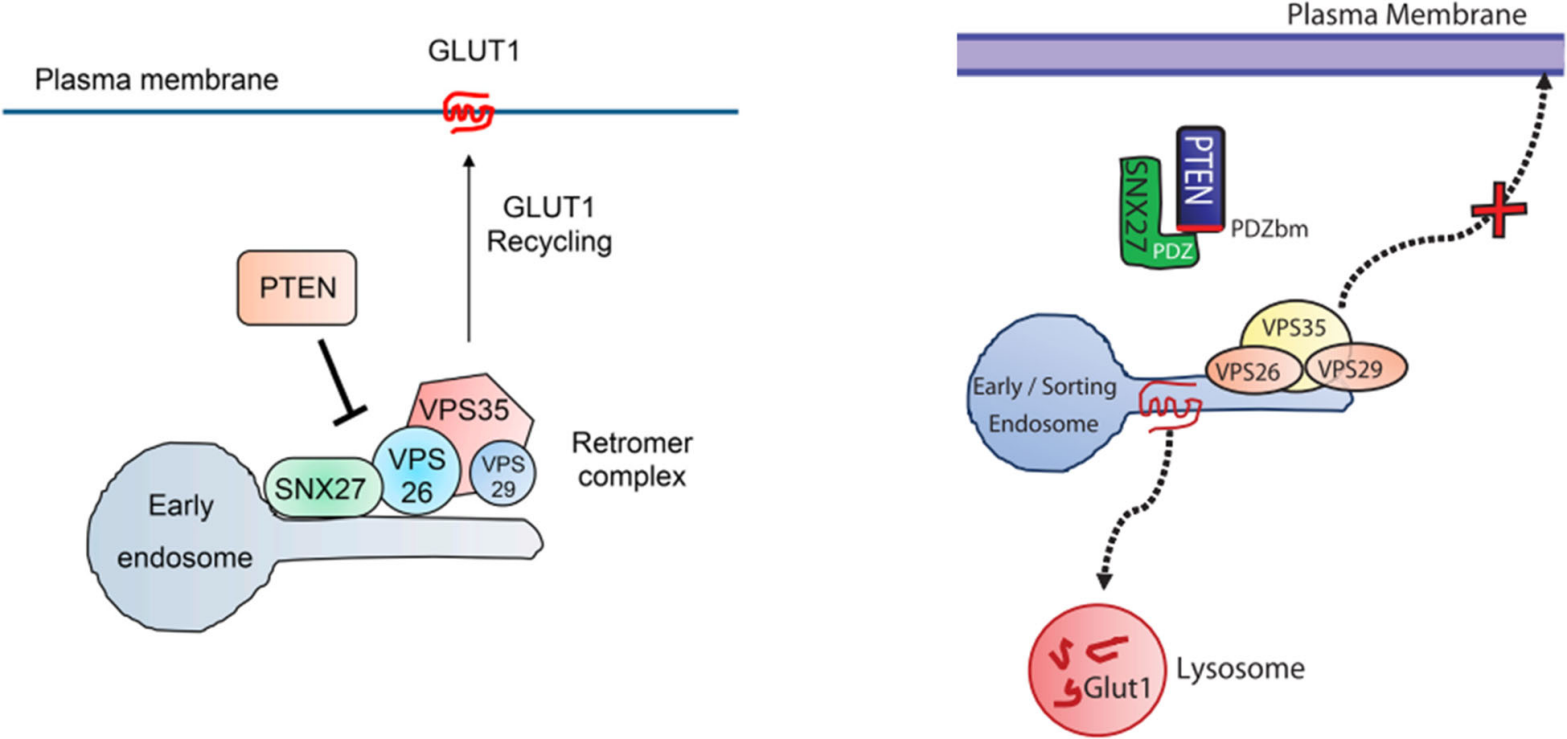
SNX27 mutants rescue PTEN-mediated repression of GLUT1 levels and glucose uptake

PTEN (phosphatase and tensin homolog deleted on chromosome 10) is known as a protein and lipid phosphatase which is a tumor suppressor and a negative regulator of cell growth and metabolism. This gene mutated frequently in many advanced human cancers [52]. PTEN expression and activity may be affected by intragenic mutations or epigenetic silencing and post-translational modifications. Histone de-acetylation is one factor of epigenetically silenced process effecting on PTEN expression. Researches in this area indicated that the inhibition of histone de-acetylase, could rescue PTEN expression and down-regulate the AKT pathway and glucose uptake [53]. No expression of a tumor-suppressor gene, phosphatase and tensin homolog (PTEN), a phosphatase that blocks the PI3K/AKT signaling pathway, has also been implicated in the development of FTC. Studies explored the association of PTEN plasma levels with sporadic PTC and their potential ability as diagnostic circulating biomarkers. The detection of PTEN promoter hypermethylation in about 50% of PTCs and almost 100% of FTCs suggests that it might be involved in the thyroid tumorigenesis [54, 55]. The signaling pathways involved in the glucose uptake in thyroid cancer cells have cited in only numerable studies. Genetic manipulations indicated that PTEN as an Oncosuppressor impress on expression of GLUT1 and glucose uptake in thyroid cancer cells. Loss of expression of the PTEN can switches off the AKT pathway and it is associated with probability of rapid thyroid cancer detection by FDG–PET. PTEN attaches SNX27 and prevent its accessibility to the VPS26 retromer complex, so blocks recycling of the glucose transporter GLUT1 to the plasma membrane, this process leads to impaired cellular glucose uptake (Fig. 1) [56, 57]. PTEN can also effect on glucose metabolism by dephosphorylation of the insulin receptor substrate-1, thus inhibit the insulin and insulin growth factor signals that also associated with glucose metabolism [53].

**Fig. 2:**
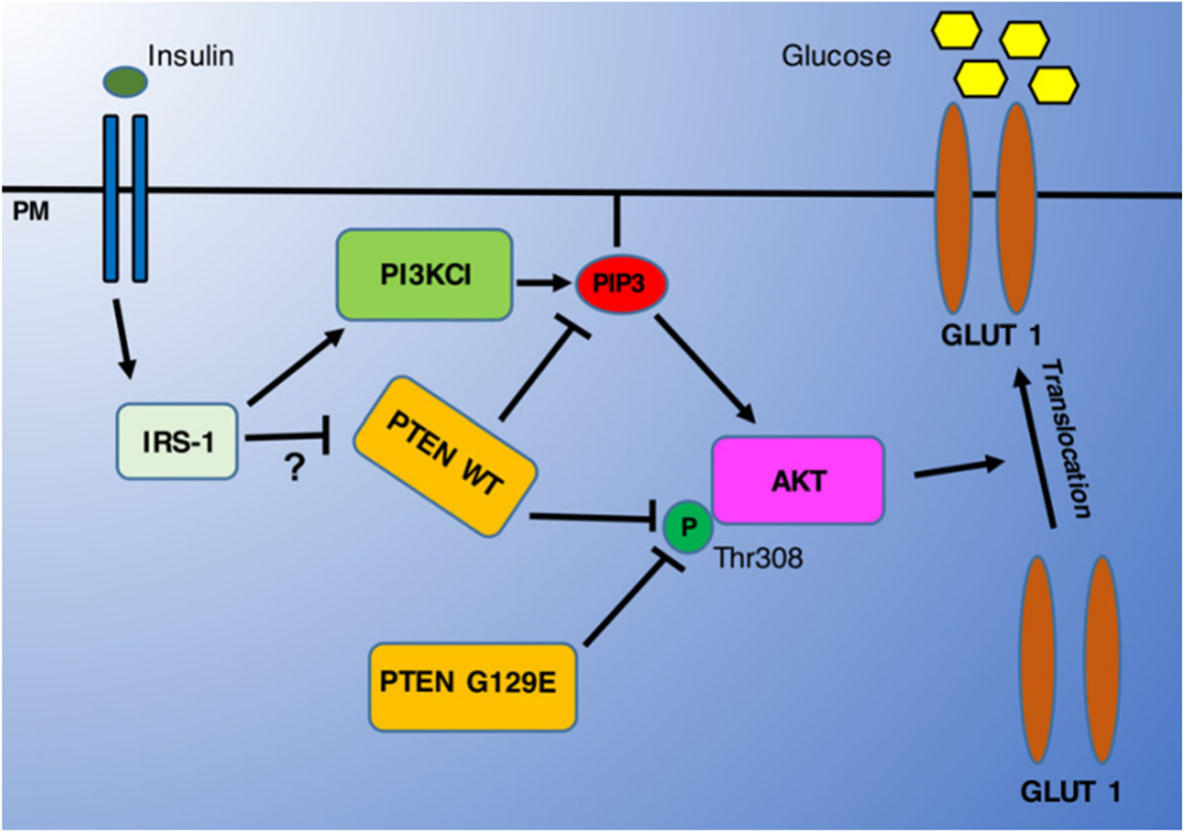
Insulin and Growth Factors can trigger the phosphorylation of AKT via activation of the PI3KC1 pathway that provides the needed PIP3. Active AKT can then promote the translocation of GLUT1 onto the plasmamembrane to effect the uptake of glucose. PTEN can switch off the AKT pathway by dephosphorylating PIP3, through its lipid-phosphatase activity, and by directly interacting and dephosphorylating AKT at the Thr308 position, through its protein-phosphatase activity

The PI3k class I (PI3KC1)-AKT pathway and of the AKT downstream effector AS160 (a rab GTPase activator) pathway are involved in the cell surface exposure of GLUT1 in thyroid cancer cells [58, 59]. The lipid phosphatase activity of PTEN is the determinative factor for inhibitory action of PTEN on AKT pathway which antagonizes the activation of the AKT pathway. This part can reduces the availability of Phosphatidylinositol (3,4,5)-trisphosphate (PIP3) which is the phosphate donor for the phosphorylation of AKT. This leads to prevention of GLUT1 expression on the plasma membrane and finally anti-cancer function. It is unrevealed whether the protein phosphatase activity of PTEN also has any effect on PI3K activity and regulation of GLUT1 on the plasma membrane (Fig. 2) Phadngam S. and et al. has been reported that PTEN can regulate AKT pathway also through the protein phosphatase activity. Actually, mutants that loose the lipid phosphatase activity while keeping the protein phosphatase activity could inhibit the AKT pathway [53].

#### P53

Environmental, genetic and hormonal agents are the main sources of increased prevalence of human cancers [60], which among them genetic mutations represent the superlative role in carcinogenesis. Several types of thyroid cancer initiation and advancement are characterized by the gradual accumulation of somatic mutations and/or gene rearrangements with different frequencies and properties [61, 62]. Nowadays, lack of the p53 family members have been signify the pathogenesis of poorly differentiated thyroid tumors. Inactivated P53 is one of the genetic alterations discriminating anaplastic thyroid cancer from well-differentiated thyroid cancers. The p53 mutations are commonly occurred in undifferentiated thyroid tumors (50–80% in ATCs) [61, 63]. Moreover in recent studies it has been indicated that p53 genetic alterations distinguished in up to 40% of papillary thyroid carcinoma and in 22% of follicular thyroid cancer [64, 65].

PTEN and P53 have major duty in driving the plasma membrane localization of GLUT1. They are key regulators of the glucose metabolism and of autophagy, which are the most common deleted or mutated oncosuppressors found in human carcinomas [59, 66, 67]. PTEN and P53 expression can reduce glucose uptake and desire its mitochondrial oxidation, thus antagonize the Warburg effect. Moreover, PTEN down-regulates the PI3k-AKT-mTOR pathway, therefore can regulates cell proliferation. Furthermore, TP53 reduces the glucose uptake through the controlling of the expression of membrane glucose transporters and glycolytic enzymes. Activation of p53 also leads to cell cycle arrest and provoke apoptotic cell death. Therefore, either loss of PTEN or of p53 are expected to increase glucose uptake, promote cell proliferation and disrupt apoptosis. Thyroid cancer cells with the altered expression of PTEN or p53 are higher consumers of glucose. These two regulators have been demonstrated to induce tumor cells to overcome the metabolic stress caused by hypoxia and glucose depletion. Also leads to inhibition of caspase-dependent apoptosis, autophagy, promotion of cell migration and invasion [68–70].

Point mutations in p53, which occurred in its DNA-binding domain correlated with malignancy and abolished its inhibitory function on transcriptional activity of GLUTs. Among the GLUTs, GLUT1 and GLUT4 gene promoters are the dominant types which affected with P53 mutations in a dose-dependent and cell type-specific manner. It is terminated in enhanced glucose metabolism and cell energy capacity which predicted to simplify tumor cell proliferation. P53 has been indicated a prominent inhibitory effect on transcriptional activity of the GLUT4 compared to GLUT1. This may be arising from the fact that GLUT1 is a general “housekeeping” glucose transporter, whereas GLUT4 is a tissue-specific and insulin-sensitive glucose transporter [71, 72].

#### MicroRNA

MicroRNAs (miRNAs) functions in carcinogenesis are subdivided into oncogenic miRNAs and tumor-suppressive miRNAs. Onco-miRNAs are upregulated in human cancers which promote cell proliferation and inhibit apoptosis, whereas tumor-suppressive miRNAs are down-regulated in human cancer and can prevent cancer development [73–75]. MiRNAs are short, non-coding, evolutionarily conserved RNAs that bind the 3’untranslated regions (3′-UTRs) of mRNAs and act as negative post-transcriptionally regulators of gene expression [76]. Current evidences indicate that Dysregulation of miRNA expression is a usual specification of thyroid cancer invasion. MicroRNAs such as miR-146b, miR-221, and miR-222 are the predictive cause of invasive PTC compared to normal tissues [77–79]. MicroRNA-718 (miR-718) plays an antioncogenic role in PTC which identified as a crucial negative regulator of PTC cell proliferation, growth, metastatic ability, and glucose metabolism. Negatively regulation of the Akt-mTOR signaling pathway by MiR-718 can reduces PTC cell phenotypic severity. MiR-718 expression was significantly downregulates in cancer tissues as compared to normal papillary thyroid tissues. According to the detection of the impact of miR-718 expression on levels of PDPK1, p-Akt, Akt, p-mTOR, it has been reported that p-Akt and p-mTOR were reduced after treatment of TPC-1 and K1 PTC cells with miR-718. This concluded that microRNAs can effect negatively on main steps of Akt-mTOR signaling pathway. This follows regulation of PTC cell proliferation, migration, and invasion. It has been demonstrated that Akt-mTOR signaling pathway has a crucial function in tumor cell glucose metabolism and phenotypic severity. MiR-718 overexpression has significant effect on reduction of energy production in PTC cells. Altogether, these results propose that microRNAs such as miR-718 are the negative regulator of metabolic activity in thyroid cancer cells [80, 81].

**Fig. 3:**
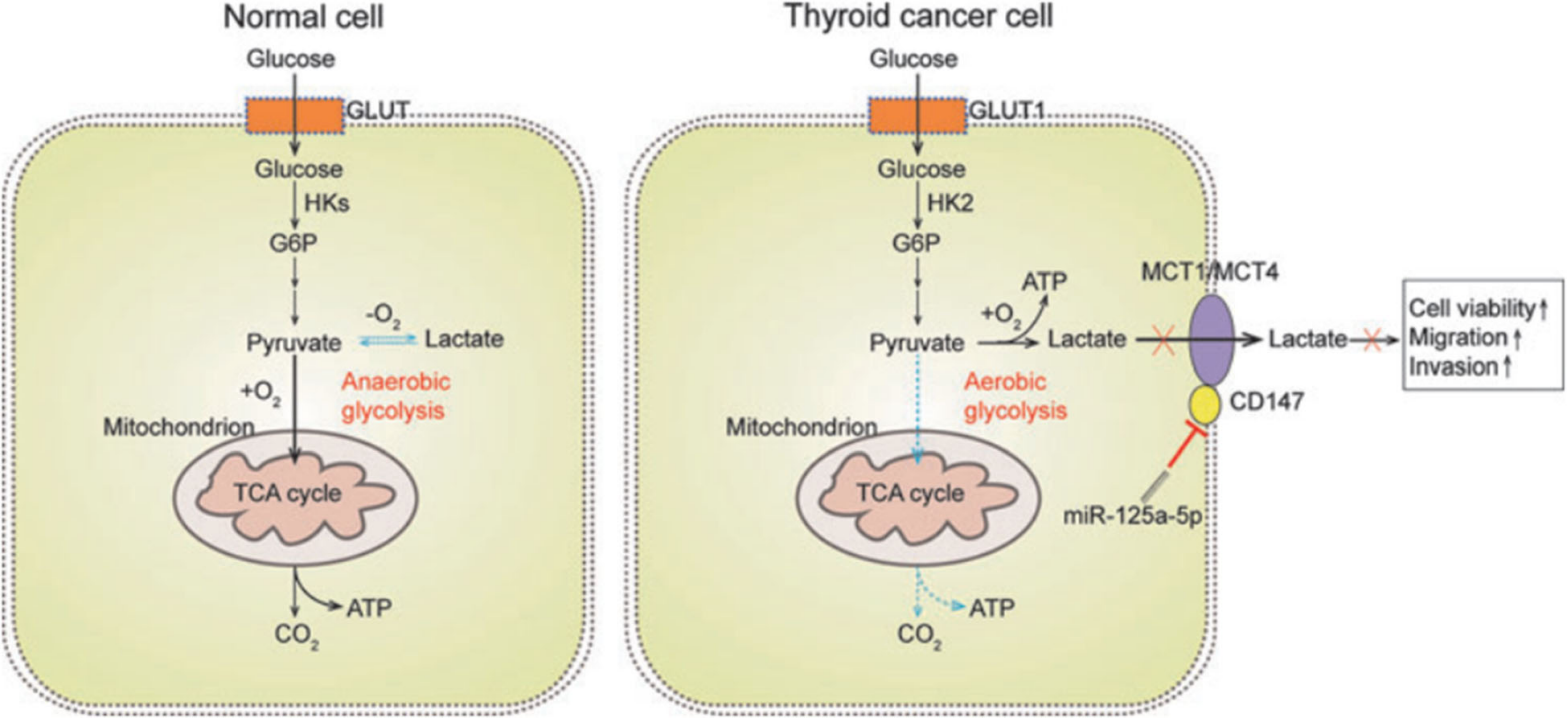
Metabolic differences between normal and TC cells. Normal cells primarily metabolize glucose to pyruvate for growth and survival, followed by complete oxidation of pyruvate to CO2 through the tricarboxylic acid cycle. TC cells convert most glucose to lactate, regardless of the availability of O2, through the overexpression of some special glucose metabolism-related proteins, including GLUT1, HK2, MCT1, and MCT4, which divert glucose metabolites from energy production to anabolic process to accelerate cell proliferation, migration and invasion. miR-125a-5p inhibits the synergistic effect of CD147 on lactate transporters (MCT1/MCT4) and reduces the viability, migration, and invasion of thyroid cancer cells, which were increased by aerobic glycolysis

MiR-125a-5p have emerged as a potent tumor suppressor and regulator of glucose metabolism in several kinds of cancers, mainly in thyroid carcinoma [82]. Since lactate is the final product of glycolysis in cancer cells and can easily be quantified using a simple enzymatic reaction, so the detection of extracellular lactate levels is an indicator of the glucose metabolism rate. MiR-125a-5p blocks lactate production, cellular ATP supply, and glucose consumption of thyroid cancer cells leading to the inhibition of glycolysis, reduction of cell migration and invasion. It is considered that the miR-125a-5p/ CD147 axis probably has an important function in the aerobic glycolysis of thyroid cancer cells. Because GLUT1, HK2, MCT1, and MCT4 are critical glycolysis-related proteins and their expression levels are significantly regulated by the miR-125a-5p/CD147 axis (Fig. 3) [83, 84].

#### Iodide

Iodine assumes an effective function in the modulation of thyroid cells activity. The number of glucose carriers in the plasma membrane can be decreased by the autoregulation performance of iodine. In fact, it influences glucose metabolism not only by effecting on the oxidation pathway, but also through an inhibitory effect on the rate-limiting glucose-facilitated transport system. Iodide is able to inhibit TSH induced stimulation of glucose transport. A role for thyroid hormone in iodine autoregulation has also been suggested, but T3 and T4 did not inhibit glucose transport. Iodide inhibits the Vmax of glucose transport without any apparent effect on the Km. This results means that iodine does not affect the affinity of the glucose transport system, but can decrease the number of available carrier sites. Malignant cells and rapidly dividing cells show a high rate of glucose transport activity. Consequently, the inhibitory effect of iodine on glucose transport in thyroid tissue may be relevant both in physiological and in pathological conditions, both at the level of intermediary and nucleic acid metabolism [85].

**Fig. 4:**
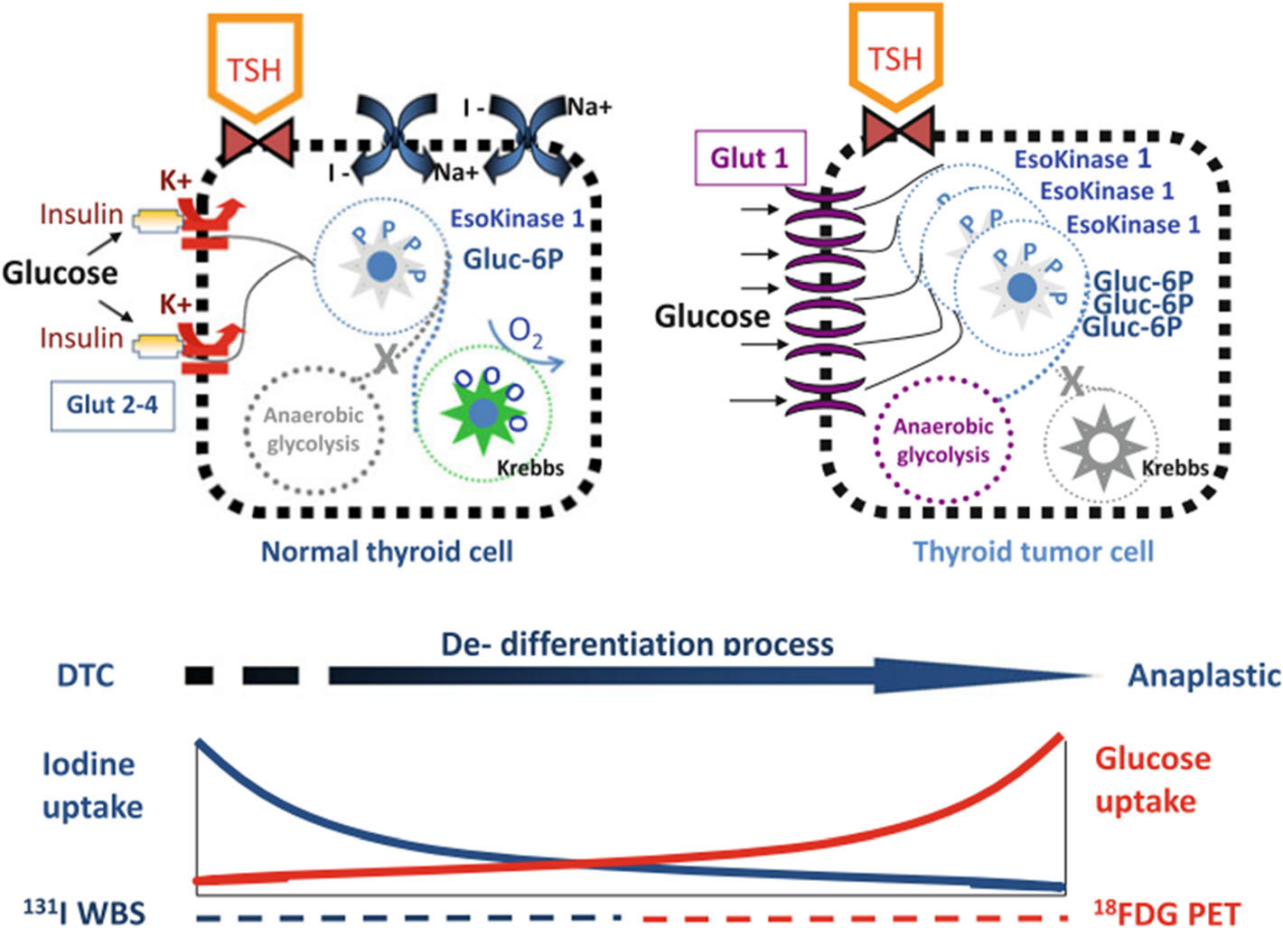
Molecular basis of dedifferentiation imaging (flipflop phenomenon)

Poor differentiation in thyroid cancers is associated with the upregulation of GLUT1, allowing cells to show more malignant biological behavior. Progressive dedifferentiation of follicle cell-derived thyroid cancer cells is tightly accompanied with a loss of iodine. Furthermore, it was considered that thyroid malignancies simultaneously become more eager for nutrients such as glucose, during dedifferentiation. This inverse relationship between iodine concentration and glucose uptake (measured by ^18^FFDG PET/CT) was designated as the flip-flop phenomenon (Fig. 4). This phenomenon is observed not only in different patients but also in different tumor sites in one patient [18, 20].

#### BRAF

BRAF (the gene for the B-type Raf kinase) is a cytoplasmic serine-threonine protein kinase which represent an essential function in thyroid oncogenesis. Detection of BRAF mutation as a common alteration in thyroid cancer is important knowledge. BRAF mutation may be an initiator in transition of papillary thyroid cancer to anaplastic thyroid cancer [86]. The differentiated thyroid cancers with a BRAF mutation indicate a higher expression of glucose transporter 1 than those with wild-type BRAF. This finding demonstrates that tumors with BRAF genetic variants may show a higher 18F-FDG uptake. It has been reported a correlation between mutated BRAF and MAPK downstream activation. This relationship targets c-Myc, HIF-1a which leads to increased glucose metabolism [87]. The occurrence of the BRAF V600E mutations may contribute to the initiation of the glycolytic phenotype was which correlated with GLUT1 overexpression. In this condition GLUT1 is the target of RAF/MEK/ERK activated pathway. This correlation leads to growth advantages in cancer cells [88, 89]. BRAF inhibitors and MAP/ERK kinase inhibitors hold great promise in the treatment of cancers associated with BRAF mutations [90].

### Positive regulators

#### HIF-1

Hypoxia is a critical hallmark of aggressive malignancies that promotes the malignant phenotype and the activation of physiological adaptation of cancer cells. This promotes tumor cell survival and disease progression. Examples of such adaptations for cancer cells include the glucose transporter 1 gene (GLUT1), which increases glucose uptake for glycolysis [91]. According to many evidences, HIF-1a signaling is upregulated in many tumors leading to tumor metastasis, poor patient prognosis and tumor resistance therapy. It is imperative to say that HIF-1a signaling is stimulated not only by lowered oxygen tension but also by oncogenic stimulation through aberrant growth factors or the loss of tumor suppressors [92]. HIF-1a protein levels and reporter gene activity upregulation is equaled with increased levels of the GLUT1. The expression of HIF-1a in FTC-133 cells bearing PTEN mutation is increased. There is an important correlation between PI3K/AKT and HIF-1a, which may have specific connection with disease progression in thyroid cancers [18].

There is no trace of HIF-1α expression in normal thyroid tissue but on the other hand in the most aggressive dedifferentiated thyroid cancers its expression is high. In PTC, MTC, and FTC, HIF-1 overexpression has been connected with a poor prognosis and distant metastasis [15, 92]. Thyroid hormones can activate both the PI3K and MAPK-signaling cascades. Moreover, thyroid hormones have the duty of directly regulation of expression of HIF-1α by stimulation of these signal transduction pathways. According to researches a TRβ mutant (TRβPV/PV) interacts with the PI3K-regulatory subunit p85α fallowing enhanced PI3K signaling, promoting thyroid carcinogenesis which may lead to increased HIF-1α signaling [92].

HIF-1 can participate in warburg phenotype through enhancing the glycolysis process. It is done not only by activation of all glycolytic enzymes transcription, but also by increasing their affinity for substrates [83]. In addition, HIF-1 also increases GLUTs expression and decreases mitochondrial metabolism which may be critical for inhibition of ROS production and protecting cancer cells from death [93, 94]. HIF-1 is suggested to contribute to the Warburg effect by stimulating a number of genes that mediate glycolysis [95, 96] including GLUT1 and GLUT3 which possess hypoxia-response elements (HRE) in their promoters [97, 98]. Moreover, HIF- 1 is directly regulated the expression of all 12 enzymes necessary for glycolysis such as the hexokinase II (HKII), phosphoglycerate kinase (PGK), glucose-6-phosphate dehydrogenase (G6PDH), and lactate dehydrogenase A (LDH-A), or glucose and lactate transporters, such as GLUT1, GLUT3 and the monocarboxylate transporter 4 (MCT4). These factors have been overexpressed in thyroid cancers. Overall, these findings proposed that some cells within thyroid tumors shift energy production by increasing glycolysis and decreasing mitochondrial function, thus exhibiting the Warburg phenotype [99]. The tight association of HIF-1α with metabolic pathways may be a pleasant target for better therapy of thyroid cancers [91].

#### PI3K/AKT

The phosphatidylinositol 3-kinase (PI3K)–Akt pathway is a member of a family of lipid and protein kinases activated by growth factors, which is responsible for regulating growth and survival processes [100]. The association of PI3K/ Akt pathway with thyroid cancer was initially proposed by propensity of patients with Cowden’s syndrome to develop thyroid cancer. Akt phosphorylates a plenty of downstream cytoplasmic and nuclear mediators hence it is responsible for regulation of multiple processes such as glucose metabolism. PI3K/Akt high expression seems to associate with worse survival of various types of cancers. Although the PI3K/Akt pathway has a significant function in endocrine tumors, it is not well studied in this type of tumors than in others [101]. PI3K/Akt pathway is a mediator of the increased glucose uptake and overexpression of GLUTs in cancers and also involves in stimulation of glucose transport in normal insulin responsive tissues to increase the rate of glucose uptake [102]. PTEN as a tumor suppressor has the task of inhibition of PI3K/AKT pathway. Loss of this inhibitor can result in enhanced PI3K signaling leading to carcinogenesis. Akt high activation without any suppressor may be the cause of the increased glucose uptake in cancers. Akt, a serine/threonine kinase downstream of PI3K, is involved in the mediation of the Warburg effect and induction of GLUTs expression including GLUT1, GLUT3 and GLUT5 [1, 100, 102, 103]. Also it acts as a regulator of GLUT4 translocation around plasma membrane resulting in facilitation of glucose transport [102].

The effect of oncogenes on cell metabolic shift for maintaining of cell proliferation is the major aspect in thyroid tumors [43]. In many tumors PI3K/AKT pathway activation may be followed by mutations occur in RAS [104] which results in increased glycolysis flux [51, 105]. The PI3K/AKT pathway is so impressive in process of translocation of cytoplasm GLUT1 to the plasma membrane in thyroid cells [15, 31]. According to the Catalogue of somatic mutations in cancer, the occurrence of mutations in the PI3K/Akt pathway are more likely in follicular and anaplastic thyroid cancers but on the other hand are less frequent in papillary thyroid cancer. Animal models have proofed elevated PI3K/Akt signaling in thyroid carcinogenesis. Akt deficiency significantly effects on reduction of the thyroid cancers incidence. Also there is growing evidence for AKT activation in human thyroid carcinoma [95, 106, 107]. In current clinical trials, many drugs targeting the PI3K/Akt signaling pathways have been studied in phases-I to III clinical trials. Among these drugs, temsirolimus and everolimus have been detected to have significant effect in thyroid cancers therapy [108].

#### TSH

TSH stands for thyroid stimulating hormone is the major factor for the regulation of function and metabolism of normal thyroid cells. Its stimulation increases glucose metabolism to amplify iodide transport and thyroid hormone (T3 and T4) synthesis [15]. Enhanced glucose uptake in tumor cells may be the reflection of alterations in gene expression or increased translocation to the cell surface. According to investigations thyroid cells represent increased glucose uptake following the thyroid-stimulating hormone activation. But expression of GLUT genes does not appear to be significantly affected by TSH suggesting that TSH change level of glucose uptake by altering GLUT localization/ translocation rather than through increased GLUT gene expression [27]. The cellular uptake of 2-deoxy-D-glucose and of the glucose transport tracer 3-O-methyl-D-glucose, both labelled by carbon-14, is significantly enhanced by TSH in the rat thyroid cell line FRTL-5. Also it is indicated that increased glucose transport can be the reason of an increased translocation of Glut-1 towards the thyroid cell surface. This results may be as an explanation of increased FDG uptake by high TSH [109].

**Fig. 5:**
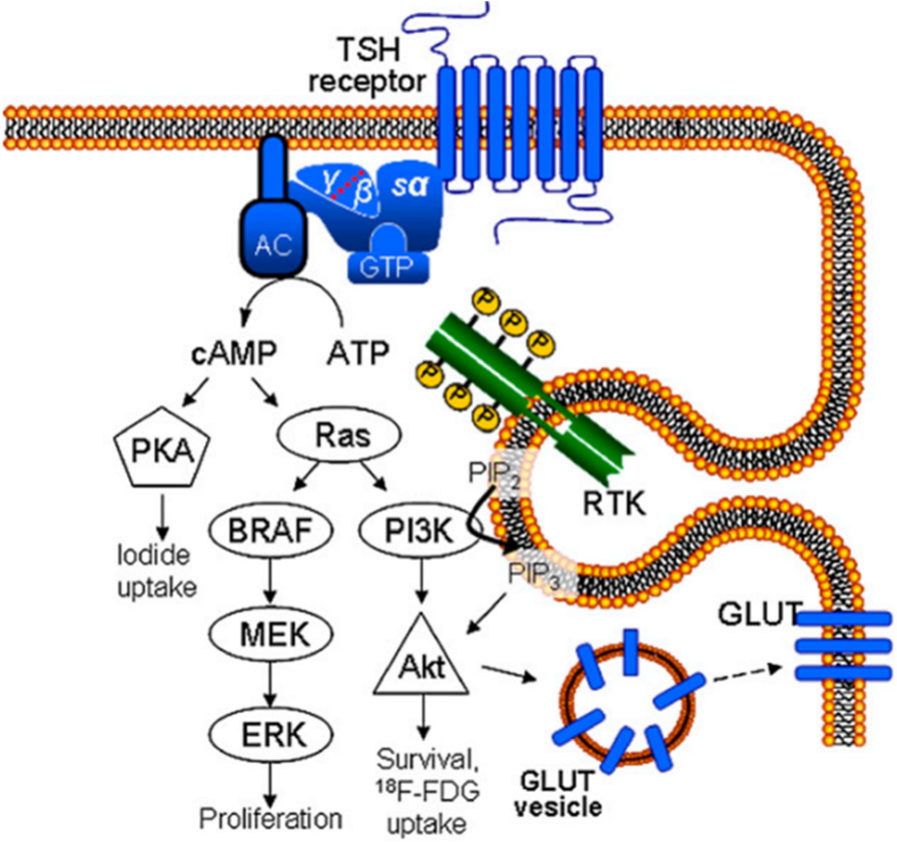
Putative scheme of signal transduction in thyreocyte according to Riesco-Eizaguirre and Santisteban and Rivas and Santisteban [34, 41]. TSH-stimulated 18F-FDG uptake is mediated via adenylate cyclase (AC) and cAMP, ras, PI3K, and Akt. Iodide uptake is regulated by PKA. Mitogenactivated protein (MAP) kinase pathway involving B-type Raf (BRAF), extracellular signal–regulated kinase, and mitogenactivated protein kinase kinase is supposed to steer cell proliferation. The Gly12Ser point mutation of K-ras oncogene as detected in ML-1 cells results in constitutive activation of K-ras-PI3K-Akt pathway in this cell line, leading to enhanced glucose transport and use

In normal thyroid tissue, glucose uptake can be stimulated by TSH [110]. Stimulated TSH influences on the adenylate cyclase, which produced an increased concentration of cAMP. The results is the enhancement of glucose metabolism in the well-differentiated rat cell line FRTL-5. The TSH-induced increase of18F-FDG accumulation depend on phosphatidylinositol-3-kinase (PI3-kinase) in FRTL-5 cells. Glucose uptake in the thyroid carcinoma cell is regulated by TSH or cAMP. It depends on PI3-kinase activity resulting from the mutated K-ras oncogene. The amount of glucose uptake influenced from TSH in ML-1 cells were compared with those of a FRTL-5 cell lines. This variability depends on the clinically heterogeneous behavior of various tumor phenotype and differentiation degrees of tumor cells. Increased activity of the second messenger PI3-kinase potentially caused by oncogenes such as mutated Ras is the reason of glucose uptake in dedifferentiated thyroid cancer which signify the pathogenesis of thyroid malignancies (Fig. 5) [51, 104, 111].

TSH exerts its effect on FDG uptake via the TSH receptors in a time- and concentration-dependent manner. In well-differentiated thyroid carcinomas, most of which are still TSH-sensitive, Glucose uptake is increased at high levels of TSH. It has been found that the expression level of the TSH receptor messenger RNA in thyroid tumors is related to the level of differentiation and that poorly differentiated thyroid carcinomas may lack TSH receptors completely [110, 112]. Furthermore, even when expressing the TSH receptor, cancerous tissue may react differently to TSH stimulation than normal tissue since somatic mutations of the TSH receptor gene and also other alterations of the CAMP cascade have been detected in thyroid tumors. Therefore, not all thyroid carcinomas would be expected to increase FDG accumulation on TSH stimulation to the same degree as normal thyroid tissue [109]. The study of correlation between FDG uptake and TSH levels are of clinical importance and may cause serious misinterpretations in the therapy investigations [111].

#### c-Myc

**Fig. 6:**
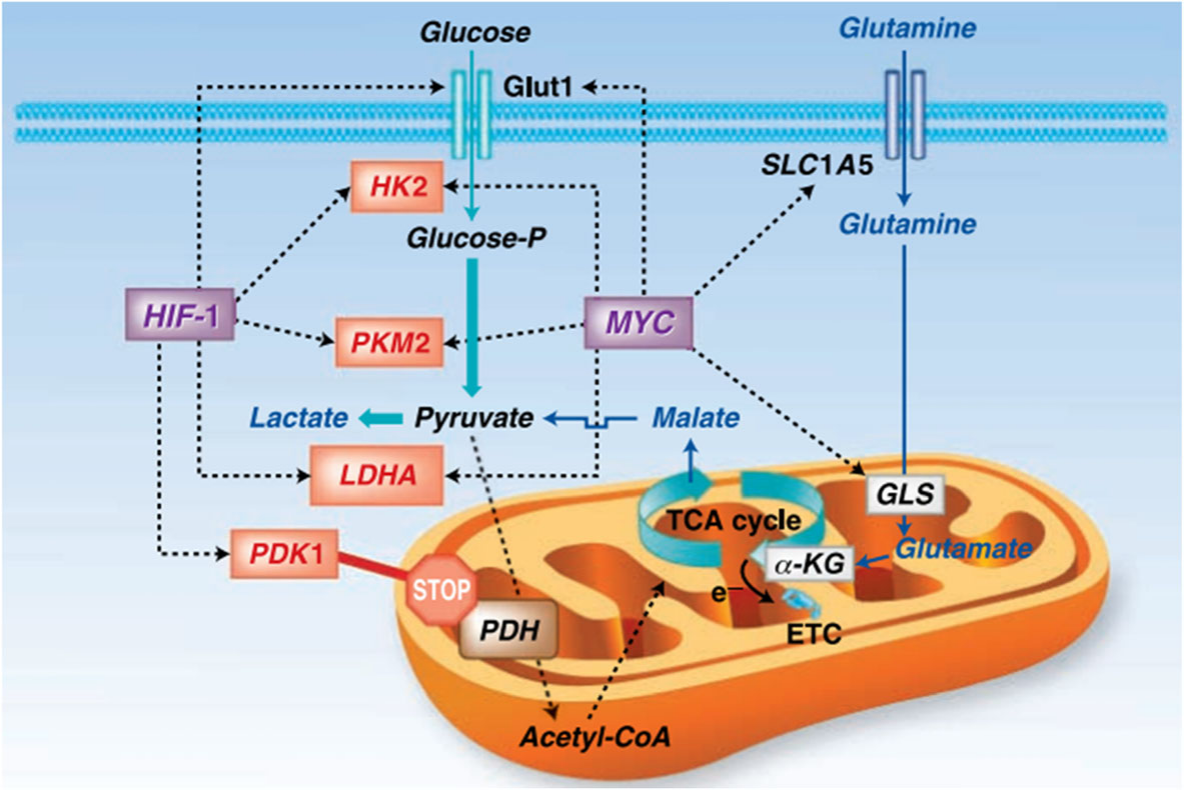
Myc and HIF-1 regulate glucose metabolism and stimulate the Warburg effect. Myc and HIF-1 are depicted to regulate (dotted lines) genes involved in glucose metabolism (glucose transporter Glut1, HK2, PKM2, LDHA, and PDK1), favoring the conversion of glucose to lactate (glycolysis). Myc is also depicted to stimulate glutamine metabolism through the regulation of transporters (SLC1A5) and glutaminase (GLS)

**Fig. 7:**
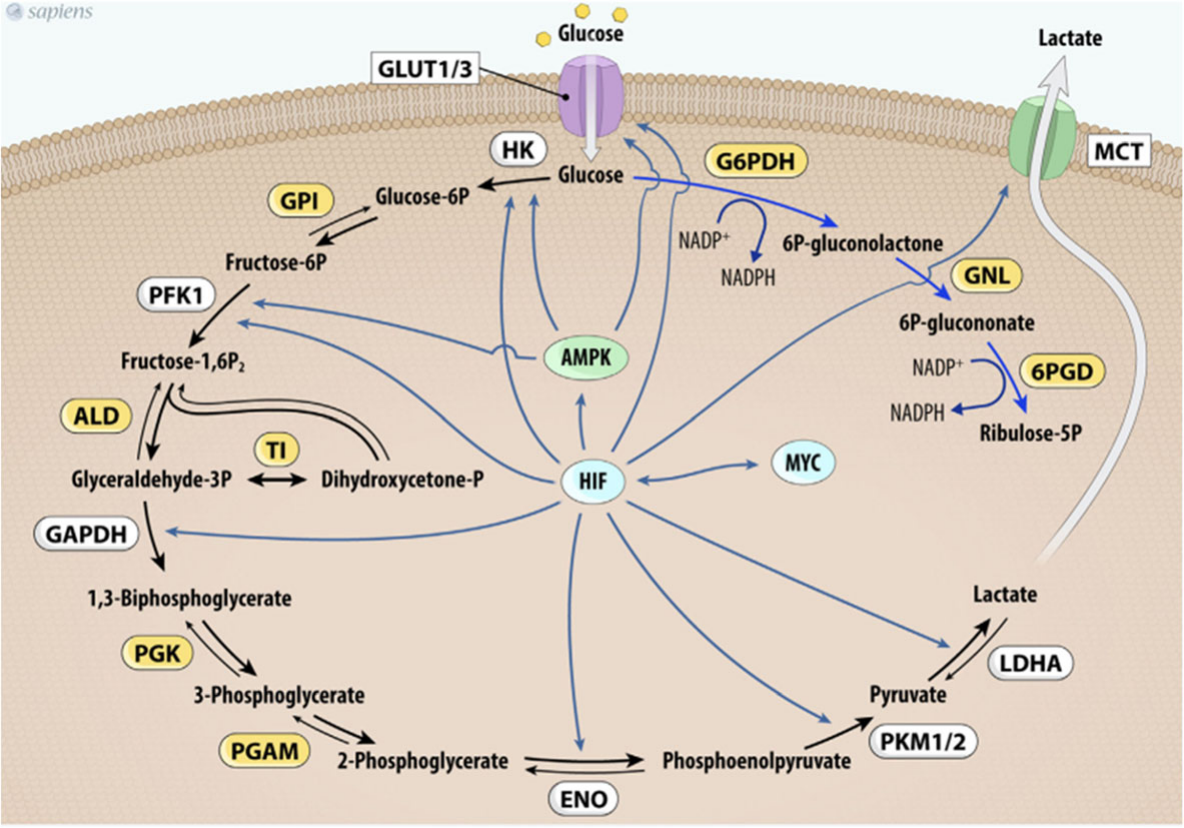
MYC and HIF-1 regulate glucose metabolism. MYC and HIF-1 are described as important regulators of key genes (in white) involved in glucose uptake and glycolysis pathway control. Abbrevations: Glut1/3, glucose transporter 1 or 3; HK, hexokinase; GPI, glucose phosphate isomerase; PFK-1, phosphofructokinase 1; ALD, aldolase; TI, triose phosphate isomerase; GAPDH, glyceraldehyde 3-phosphate dehydrogenase; PGK, phosphoglycerate kinase; PGAM, phosphoglycerate mutase; ENO, enolase; PKM1/2, pyruvate kinase isoforms 1 and 2; LDHA, lactate dehydrogenase A; G6PDH, glucose 6-phosphate dehydrogenase; GNL, gluconolactonase; 6PDG, 6-phosphogluconate dehydrogenase; MCT1, monocarboxylate transporter-1; HIF-1, hypoxia-inducible factor-1

The c-MYC proto-oncogene is an obvious cause of cancers. Glucose metabolism genes were demonstrated to be directly regulated by Myc. Chief among these are the glucose transporter GLUT1, hexokinase 2 (HK2), phosphofructokinase (PFKM), and enolase 1 [113–115]. Glucose metabolism inhibitors targeting MYC have recently shown the ability to inhibit GLUT-1, LDH-A, and MCT1 expression in cancer cell lines, along with decreased MYC activity, resulting in reduced cell proliferation and tumor growth [116]. Myc regulates genes involved in the transportation of glucose, its catabolism to trioses and pyruvate, and ultimately to lactate. Because glycolytic genes are also directly responsive to HIF-1, the collaboration between Myc and HIF was documented in many tumors containing genetic alterations [16, 116, 117]. Myc could stimulate glucose oxidation and lactate production in normoxia. Under hypoxia, Myc interplay with HIF-1 to induce Phosphoinositide-dependent kinase-1, suppress mitochondrial respiration and finally preferring anaerobic glycolysis (Fig. 6) [118]. In addition to thyroid cancer, c-Myc overexpression has been identified in various cancers and it upregulates the expression of genes involved in glucose metabolism. The c-Myc is a factor that links cellular metabolism shifts to carcinogenesis. The first connection found between c-Myc and glycolysis was the effect of this factor on positive regulation of the enzyme that converts pyruvate from glycolysis to lactate. Furthermore, glucose transporter-1, Hexokinase 2, phosphofructokinase and Enolase 1 were also known as c-Myc targets (Fig. 7) [118].

#### AMPK

Reactive oxygen species (ROS) as upstream signals of AMP kinase (AMPK) protein can change cell metabolism and increase Warburg effect by upregulation of AMPK. AMPK is a metabolic stress-sensing cytosolic enzyme that activated in shortage of energy for regulation of metabolism and cell growth [119]. In contrast, the production of ROS is not under the control of AMPK. It is proved by the inhibition of AMPK phosphorylation and evaluation of ROS production in this condition. AMPK indicates essential role in stopping points of the cell cycle and has a strong antigrowth effect in various cancer cell lines. In thyroid glands, it presents a serious physiological role in thyroid iodide uptake in vitro and in vivo and could be involved in strong aggressiveness of thyroid cancer. In the activated state of AMPK, it tends to upregulates energy-producing pathways and restore intracellular ATP levels, conversely shuts down the processes of consuming energy. AMPK activation also increases glucose uptake mainly in the first step of glycolysis pathway in both nontumoric cells and papillary thyroid cancer cells. There is evidence that AMPK can increases glucose uptake in thyroid cells without any dependence of TSH by increasing both GLUT 1 expression and hexokinase (HK) activity. It clearly demonstrates that AMPK involves an alternative pathway for regulating glucose uptake by thyrocytes. Enhancement of glucose transport into cells and higher glucose phosphorylation rate are the two pathways account for glucose metabolism modulation. There are three kinases including liver kinase B1 homolog (serine/threonineprotein kinase LKB1), calcium/calmodulin-dependent protein kinase kinase (CaMKK), or TGF-beta-activated kinase 1 (TAK1) which might play a role in the regulation of glucose uptake by thyrocytes. The ability of AMPK to modulate glucose metabolism might be useful for future identification of novel pathways involved in the regulation of thyroid function [32, 120]. The inhibition of AMPK activity can stimulates the Warburg effect in tumor cells. Data from AMPK deficient models demonstrate that mTOR is dramatically upregulated. Also in this condition HIF-1 elevates HK2 and Glut1 expression and increases glucose consumption by tumor cells. The activation of AMPK seems to be involved in the glucose metabolism that is observed in some PTC cells. The expression of the active phosphorylated form of AMPK in PTC tumor cells sample is greater than that in non-tumor tissues sample. Finally, novel experimentations are vital for elucidation of the role of AMPK in human thyroid cancer, especially in metabolic control aspects including cell growth, apoptosis, and survival [121–123].

## Conclusion

Cancer historically definition described it as an abnormal cell proliferation but recent evidences proved that cancer is indeed a kind of metabolic disorder. It seems that in the near future, metabolomics studies, besides conventional methods will be used for diagnosis and differentiation of different types of thyroid cancers and will most likely introduce altered metabolic pathways as therapeutic targets [124]. The fast prevalence grade of thyroid cancer is higher than that of any other type of cancers in some countries [125]. Thyroid cancer cells show a wide complexity in metabolic pathways which give the ability of reprograming glucose metabolism upon nutrient deprivation conditions in hypoxic tumor microenvironment. Malignant cells indicate metabolic modifications till obtain more energy to acquire the capability to sustain proliferative signaling. To this purpose, in more aggressive thyroid cancer, metabolic feature changes such as increased glucose uptake can be observed. Perception of the mechanisms by which glucose is transported into normal and pathologic thyroid tissues could provide effective insights into diagnosis and treatment of thyroid cancer therapies [27, 126]. Anticancer treatment is based on two main aspects. One is the traditional aspect based on conventional chemotherapy addressed nonspecifically against general cell processes. The other is new approaches stands on the application of molecular targeted therapy, it includes drugs designed to inhibit specific components of deregulated signaling pathways in cancer [127]. Deregulation of cellular metabolism as a great hallmark of cancer may reflect the presence of alterations in different signaling pathways. The metabolic shift suggests a survival privilege for tumor cells [128]. In spite of high frequency of different biomarkers discovered for thyroid cancers, just few of them could be applied in clinical cases. In many cases using each of these molecules alone may not be effective, therefore, combination of two or more biomarkers could be of great assistance in diagnosis and prediction of thyroid cancer [129]. In this review the regulation of GLUTs by key proliferation and pro-survival pathways including PI3K-Akt, HIF-1, MicroRNA, PTEN, AMPK, BRAF, c-Myc and p53 pathways is discussed.

As glucose uptake into cancer cells is the rate-limiting step in glycolysis, targeting GLUTs by blocking their glucose transport channel with small molecules should be a viable mechanism of nutrient deprivation in tumors [130]. The principium for targeting glucose transporters in cancer has been emphasized recently [1]. Glucose transport inhibitors have been shown to be promising anti-cancer agents that warrant further basic science and clinical investigation. As we have accumulated more information about cancer metabolism, so we will have the capability of developing more beneficial anti-cancer-metabolism drugs [37]. Discoveration of method for disruption of glucose uptake via glucose transporter proteins may alter the metabolism of malignant cells, resulting in weaken tumor growth. If this hypothesis is supported, then glucose transporter proteins could become meaningful targets for the treatment of cancer [123]. Altering the balance between cancer and stromal cells or the metabolic cooperativity among different TC cell populations is a promising therapeutic strategy but still needs to be further studied. Finally, to give a therapeutic option to poorly differentiated and refractory TC, a better characterization of the metabolic phenotype of TC subtypes is clearly needed [126]. Conventional therapies such as surgical thyroidectomy and radioiodine treatment have been the main aspect of treatment for thyroid cancer, but they are often not curative. Hence, it is necessary to recast therapeutic strategies against these types of tumors for achieving modern drugs. Nowadays, the levels of expression of GLUT1 or GLUT3 may provide valuable science around the indication of aggressiveness and progression of a tumor or patient survivance. The potential use of GLUTs as targets for therapeutics is a promising area to pursue in future studies for combining therapy.

## Data Availability

Not applicable.

## Abbreviations

AMPK: 5' adenosine monophosphate-activated protein kinase
ATCs: Anaplastic thyroid cancers
AC: Adenylate cyclase
Akt-mTOR: Protein Kinase B-Mammalian Target of Rapamycin
ALD: Aldolase
AS160: A rab GTPase activator
BRAF: A human gene that encodes a protein called B-Raf
cAMP: Cyclic Adenosine Monophosphate
CD147: A transmembrane glycoprotein that belongs to the immunoglobulin superfamily
c-Myc: Master Regulator of Cell Cycle Entry and Proliferative Metabolism
ENO: Enolase
FDG–PET: Fluorodeoxyglucose-positron emission tomography
FTC: Follicular Thyroid Carcinoma
FDG: Fluorodeoxyglucose
GLUTs: Facilitative glucose transporter
GPI: Glucose phosphate isomerase
G6PDH: Glucose-6-phosphate dehydrogenase
GAPDH: Glyceraldehyde 3-phosphate dehydrogenase
GNL: Gluconolactonase
HIF-1: *Hypoxia-inducible factor 1*
HK2: Hexokinase 2
HRE: Hypoxia-response elements
HKII: Hexokinase II
K-ras: Kirsten rat sarcoma viral oncogene homolog
LDH-A: Lactate dehydrogenase A
miRNAs: MicroRNAs
mRNAs: Messenger RNA
MCT: Monocarboxylate transporter
MAPK: Mitogen-activated protein kinase
mTOR: Mammalian target of rapamycin
PKA: Protein kinase A
PFKM: Phosphofructokinase
PDK1: *3-phosphoinositide-dependent protein kinase-1*
PKM2: Pyruvate kinase muscle isozyme M2
PFK-1: Phosphofructokinase 1
(PI3K)-Akt: Phosphatidylinositol 3-kinase (PI3K)–Akt pathway
PGK: Phosphoglycerate kinase
PGAM: Phosphoglycerate mutase
PKM1/2: Pyruvate kinase isoforms 1 and 2
6PDG: 6-Phosphogluconate dehydrogenase
PTEN: Phosphatase and tensin homolog
p53: Tumor protein 53
PPP: Pentose phosphate pathway
PTC: Papillary Thyroid Cancer
PIP3: Phosphatidylinositol (3,4,5)-trisphosphate
SNX27: Sorting nexin 27
SGLT: The sodium-coupled glucose co-transporter
SLC2A: Solute Carrier 2A
TSH: Thyroid stimulating hormone
T3: Triiodothyronine
T4: Thyroxine
TC: Thyroid cancer
TI: Triose phosphate isomerase
3’-UTRs: 3’untranslated regions
VPS26: Vacuolar protein sorting-associated protein 26A
WDTCs: Well-differentiated thyroid carcinomas
