# Correction to: Regulators of glucose uptake in thyroid cancer cell lines

**DOI:** 10.1186/s12964-022-00826-2

**Published:** 2022-01-25

**Authors:** Shabnam Heydarzadeh, Ali Asghar Moshtaghie, Maryam Daneshpour, Mehdi Hedayati

**Affiliations:** 1 Department of Biochemistry, School of Biological Sciences, Falavarjan Branch Islamic Azad University, Isfahan, Iran; 2 Cellular and Molecular Endocrine Research Center, Research Institute for Endocrine Sciences, Shahid Beheshti University of Medical Sciences, Tehran, Iran

## Correction to: Cell Communication and Signaling (2020) 18: 83 10.1186/s12964-020-00586-x

Following publication of the original article [1], the authors identified an error in the author name of Maryam Daneshpour.

The incorrect author name is: Maryam Daneshpoor.

The correct author name is: Maryam Daneshpour.
